# Clinical outcomes of the immediate reapplication of small-incision lenticule extraction without adjusting the surgical parameters after suction loss

**DOI:** 10.1038/s41598-022-20403-4

**Published:** 2022-09-24

**Authors:** Byunghoon Chung, Ik Hee Ryu, In Sik Lee, Jin Kuk Kim, Tae-im Kim, Eung Kweon Kim, Kyoung Yul Seo, Ikhyun Jun

**Affiliations:** 1 Department of Ophthalmology, The Institute of Vision Research, Yonsei University College of Medicine, 50-1 Yonsei-ro, Seodaemungu, Seoul, 03722 Republic of Korea; 2 Corneal Dystrophy Research Institute, Yonsei University College of Medicine, Seoul, Republic of Korea; 3 B&VIIT Eye Center, Seoul, Republic of Korea; 4 VISUWORKS, Seoul, Republic of Korea; 5 Saevit Eye Hospital, Goyang-Si, Gyeonggi-Do Republic of Korea

**Keywords:** Medical research, Outcomes research

## Abstract

This study was to analyze the clinical outcomes of immediate reapplication of small-incision lenticule extraction (SMILE) without adjusting the surgical parameters after suction loss and to compare the outcomes with contralateral eyes that underwent uneventful SMILE. A total of 74 patients who underwent uneventful SMILE in one eye (Uneventful group) and immediate reapplication of SMILE without adjusting the surgical parameters after suction loss in the contralateral eye (Suction loss group) were included. Suction loss occurred during the posterior lenticule surface cut in 39 eyes (53%) and the cap cut in 35 eyes (47%). Surgical outcomes, including visual acuity, manifest refraction, keratometry, and corneal wavefront aberrations, were evaluated at 6 months postoperatively. The mean uncorrected distance visual acuity (UDVA), corrected distance visual acuity (CDVA), and spherical equivalent were − 0.02 ± 0.07, − 0.04 ± 0.04, and − 0.10 ± 0.46 diopters (D), respectively, in the Suction loss group and − 0.02 ± 0.07, − 0.04 ± 0.05, and − 0.19 ± 0.53 D, respectively (*P* = 0.965, 0.519, and 0.265, respectively), in the Uneventful group. Changes between the preoperative and 6-month postoperative total corneal aberrations, spherical aberrations, and horizontal and vertical coma did not significantly differ between the Suction loss and Uneventful groups. Immediate reapplication of SMILE without adjusting the surgical parameters after suction loss resulted in good surgical outcomes that were comparable with those of uneventful SMILE.

## Introduction

Small-incision lenticule extraction (SMILE) has been accepted as a safe and effective corneal refractive surgery technique for the treatment of myopia and myopic astigmatism^1,2^. During SMILE, a photodisruptive femtosecond laser is used to create a refractive lenticule inside the corneal stroma and the refractive lenticule is extracted through a small corneal incision. The surgical outcomes of SMILE are reported to be comparable to those of other corneal refractive surgery techniques, including laser in-situ keratomileusis (LASIK) and transepithelial photorefractive keratectomy (PRK)^3–6^.

To safely create the refractive lenticule and corneal incision during the SMILE procedure, a patient’s eye should be in a secure position. A vacuum is applied using a suction device to maintain the patient’s eye in a secure position. The loss of suction is an intraoperative complication of SMILE with an incidence of 0.17–4.40%^1,7–12^. Treatment options for suction loss during SMILE include immediately reapplying SMILE, reprogramming the SMILE procedure with a thinner cap, immediately converting the technique to LASIK or transepithelial PRK, and delaying the surgery^13–15^. The treatment options vary according to the surgical stage at which suction loss occurs. Further, some studies have reported comparable outcomes between cases in which suction loss occurred and uneventful cases^13,14,16^. However, another study demonstrated unfavorable outcomes in suction loss cases compared with uneventful cases^9^. Also, studies on how to manage the suction loss that occurred during SMILE are lacking.

This study aimed to investigate the surgical outcomes of patients who underwent immediate reapplication of the SMILE procedure without adjustment of the surgical parameters following suction loss and to compare these outcomes with those of contralateral eyes that underwent uneventful SMILE.

## Patients and methods

This was a retrospective observational paired-eye case series conducted at B&VIIT Eye Center, Seoul, Republic of Korea. The study protocol followed the tenets of the Declaration of Helsinki and good clinical practice. Yonsei University College of Medicine institutional review board approved the study, and the same institutional review board waived the requirement for informed consent as this was a retrospective study. All patients underwent SMILE between January 2017 and March 2019. All surgeries were performed by two experienced surgeons (IHR and ISL). Patients who underwent uneventful SMILE in one eye (the Uneventful group) and the immediate reapplication of SMILE without adjustment of the surgical parameters following suction loss in the contralateral eye (Suction loss group) were enrolled.

The inclusion criteria included aged 20–45 years, stable myopia for ≥ 1 year, a corrected distance visual acuity (CDVA) of 20/25 or better, a spherical equivalent (SEQ) refraction of − 2.00 to − 8.00 diopters (D), and refractive astigmatism of < 3.00 D. Patients with any ocular surface diseases; a history of corneal or intraocular surgery; ocular trauma, keratoconus; cataract; or collagen, vascular, or autoimmune diseases were excluded.

### Patient assessment

All examinations were performed before and 6 months after surgery. Patient evaluations included measurement of the logarithm of the minimum angle of resolution (logMAR), uncorrected distance visual acuity (UDVA), CDVA, manifest refraction, slit-lamp examination (Haag-Streit, Köniz, Switzerland), central corneal thickness (CCT), and Scheimpflug-based corneal tomography (Pentacam HR; Oculus Optikgeräte GmbH, Wetzlar, Germany). Corneal wavefront aberrations were assessed using a Pentacam HR in a 6 mm zone.

### Surgical technique

#### Uneventful SMILE (uneventful group)

SMILE was performed using a VisuMax 500-kHz system (software version 2.4.0; Carl Zeiss Meditec AG, Jena, Germany) with standardized techniques^17^. Laser spot spacing was set at 4.5 μm. The upper and lower edges of the refractive lenticule were delineated and a 2 mm incision was made at the 145° meridian. Once the upper and lower interfaces were separated, the lenticule was extracted using microforceps. The diameter of the cap was between 6.7 and 7.8 mm. The optical zone diameter was between 5.7 and 6.8 mm. The intended cap thickness was 90–135 μm. The default cap thickness was set to be 120 μm. The cap thickness was modified according to the residual stromal thickness, the amount of refractive correction, and the preoperative pachymetry. Postoperative medications included 0.5% moxifloxacin, and 1% prednisolone four times a day for 1 months.

#### Immediate reapplication of SMILE after suction loss (suction loss group)

When suction loss occurred during the posterior lenticule surface cut, the SMILE procedure was repeated from the beginning. The surgical parameters were re-entered without modification. When suction loss occurred during or after the cap was cut, the SMILE procedure was repeated from the point at which it was stopped. In these cases, the surgical parameters did not need to be re-entered into the laser control platform.

### Statistical analysis

Statistical analyses were performed using IBM SPSS Statistics for Windows, version 25.0 (IBM Corp., Armonk, NY, USA). The Kolmogorov–Smirnov test was used to confirm data normality. Two measurements were compared using the paired t-test for normally distributed data or the Wilcoxon signed-rank test for non-normally distributed data. Categorical variables were compared using the chi-square test, and linear regression analysis was used to investigate refractive predictability. All data are presented as the mean ± standard deviation. Statistical significance was set at *P* < 0.05.

## Results

A total of 148 eyes from 74 patients were included. Total number of patients during the study period was 17,996, and the incidence rate of suction loss was 0.4%. There was no other intraoperative or postoperative complication in both groups. The baseline characteristics, including age, sex, preoperative visual acuity, and refractive errors, are shown in Table 1. There were no significant differences in preoperative refractive errors, including sphere and cylinder values, CDVA, UDVA, CCT, keratometry, and corneal toricity between the Uneventful and Suction loss groups. Surgical parameters, including optical zone diameter and lenticule thickness, were also similar between the two groups. Suction loss occurred in 53% of the eyes during the posterior lenticule surface cut and 47% during the cap cut.

**Table 1:** Characteristics of eyes that underwent immediate SMILE reapplication after Suction loss and contralateral uneventful SMILE.

Characteristics	Suction loss	Uneventful	*P*
Number of eyes (right/left)	74 (53/21)	74 (21/53)	
Sex (male/female)	34/40		
Age (years)	26.22 ± 5.23 (19–39)		
**Refractive errors (D)**			
Sphere	− 3.94 ± 1.77 (− 8.25 to − 1.00)	− 4.01 ± 1.79 (− 7.75 to − 1.00)	0.813
Cylinder	− 0.70 ± 0.55 (− 2.25 to 0.00)	− 0.73 ± 0.56 (− 2.00 to 0.00)	0.766
Spherical equivalent	− 4.29 ± 1.77 (− 8.38 to − 1.25)	− 4.37 ± 1.85 (− 8.25 to − 1.13)	0.782
LogMAR CDVA	− 0.01 ± 0.03 (− 0.10 to 0.05)	− 0.01 ± 0.04 (− 0.10 to 0.20)	0.496
LogMAR UDVA	1.12 ± 0.32 (0.30–1.70)	1.11 ± 0.36 (0.30–1.70)	0.897
CCT (μm)	560.14 ± 25.99 (492–610)	559.31 ± 26.24 (491–604)	0.848
Average keratometry (D)	43.41 ± 1.22 (40.13–46.38)	43.39 ± 1.23 (40.38–46.25)	0.940
Corneal toricity (D)	1.24 ± 0.54 (0.00–2.50)	1.29 ± 0.59 (0.25–2.50)	0.538
Optical zone (mm)	6.41 ± 0.16 (6.00–6.80)	6.39 ± 0.19 (5.70–6.80)	0.707
Cap thickness (μm)	118.45 ± 6.72 (90–135)	118.72 ± 5.91 (105–135)	0.968
Lenticule thickness (μm)	91.86 ± 25.36 (48–149)	92.50 ± 26.13 (45–152)	0.881
**% of eyes in the surgical stage when suction loss occurred**			
Posterior lenticule surface cut	53	–	
Cap cut	47	–	

Results are expressed as mean ± standard deviation (range).

*CDVA* corrected distance visual acuity, *D* diopter, *logMAR* logarithm of the minimum angle of resolution, *UDVA* uncorrected distance visual acuity, *CCT* central corneal thickness.

### Visual outcomes, efficacy, and safety

**Figure 1:**
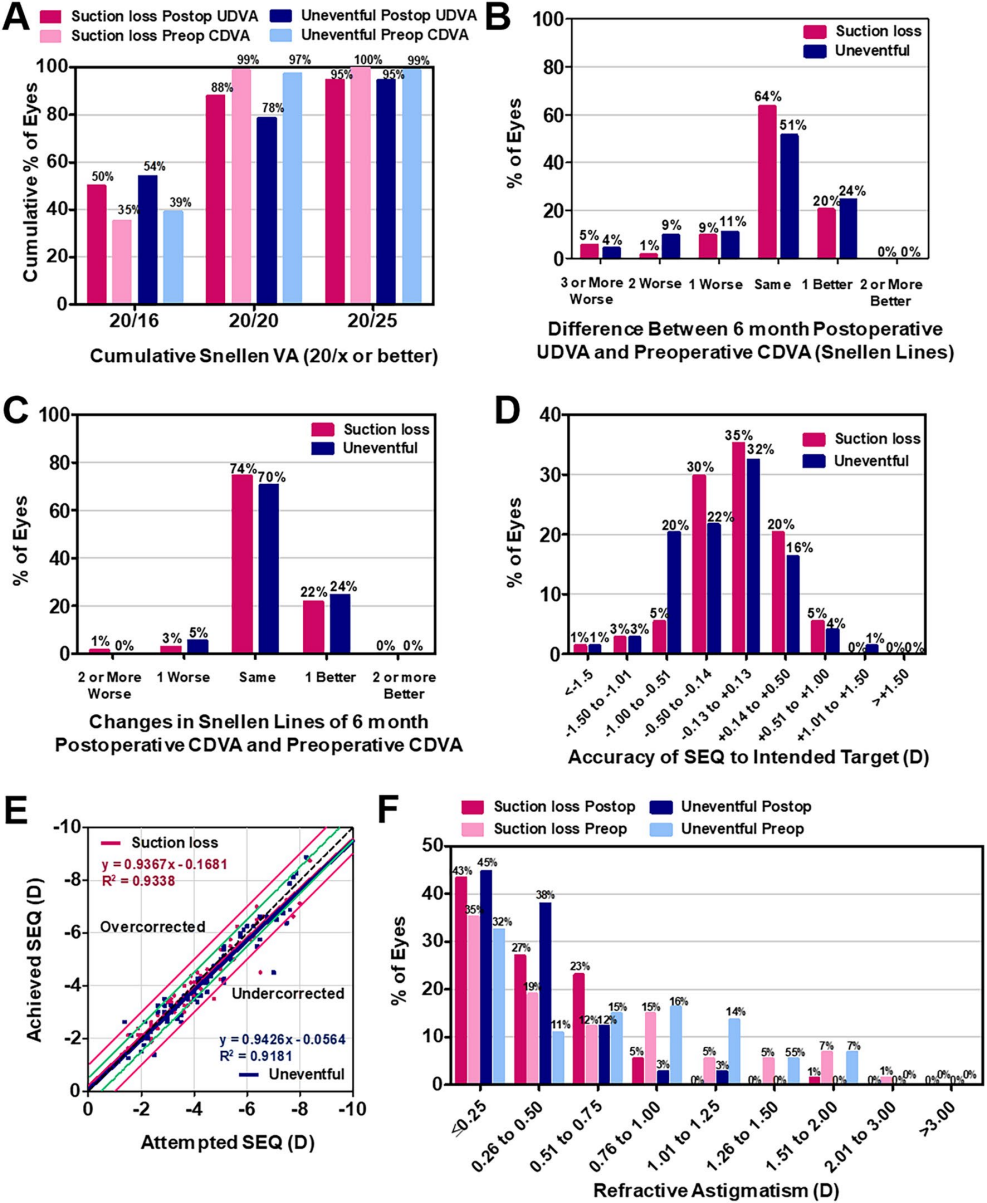
Visual and refractive outcomes after uneventful small-incision lenticule extraction (SMILE) (Uneventful group) and immediate reapplication of the SMILE procedure after suction loss (Suction loss group). (**A**) Cumulative 6-month postoperative uncorrected distance visual acuity (UDVA) and preoperative corrected distance visual acuity (CDVA). Changes in the Snellen lines of (**B**) postoperative UDVA and (**C**) CDVA relative to the preoperative CDVA. (**D**) Accuracy of the spherical equivalent refraction (SEQ) relative to the intended target and (**E**) the attempted versus achieved changes in SEQ at 6 months postoperatively. (**F**) Refractive astigmatism before and 6 months after the surgery. *D* diopters.

The mean 6-month postoperative UDVA and CDVA did not significantly differ between the Suction loss group and the Uneventful group (*P* = 0.965 and 0.519, respectively; Table 2). In total, 99% of eyes in the Suction loss group and 97% in the Uneventful group showed a postoperative 6-month UDVA of better than 20/20 (Fig. 1). Improvement in the postoperative UDVA with one or more Snellen lines relative to the preoperative CDVA was achieved in 20% and 24% of eyes in the Suction loss and Uneventful groups, respectively (Fig. 1). The mean efficacy index (ratio of postoperative UDVA to preoperative CDVA) and safety index (ratio of postoperative CDVA to preoperative CDVA) did not significantly differ between the two groups (Table 2).

**Table 2:** Comparison of postoperative 6-month visual acuity and refractive errors of patients who underwent immediate SMILE reapplication after suction loss and contralateral uneventful SMILE.

	Suction loss	Uneventful	*P*
LogMAR UDVA	− 0.02 ± 0.07 (− 0.08 to 0.30)	− 0.02 ± 0.07 (− 0.08 to 0.22)	0.965
LogMAR CDVA	− 0.04 ± 0.04 (− 0.08 to 0.05)	− 0.04 ± 0.05 (− 0.08 to 0.16)	0.519
Efficacy index	1.05 ± 0.16 (0.50–1.20)	1.06 ± 0.17 (0.60–1.20)	0.803
Safety index	1.08 ± 0.11 (0.75–1.20)	1.10 ± 0.11 (0.83–1.20)	0.294
Sphere (D)	0.14 ± 0.49 (− 2.00 to 1.00)	0.02 ± 0.53 (− 2.00 to 1.50)	0.174
Cylinder (D)	− 0.48 ± 0.31 (− 2.00 to 0.00)	− 0.44 ± 0.26 (− 1.25 to 0.00)	0.316
Spherical equivalent (D)	− 0.10 ± 0.46 (− 2.00 to 0.63)	− 0.19 ± 0.53 (− 2.50 to 1.25)	0.265
Average keratometry (D)	39.81 ± 1.73 (34.88–43.63)	39.82 ± 1.72 (35.38–44.13)	0.971
Corneal toricity (D)	0.90 ± 0.48 (0.00–2.00)	0.95 ± 0.49 (0.00–3.00)	0.496

Results are expressed as mean ± standard deviation (range).

*logMAR* logarithm of the minimum angle of resolution, *UDVA* uncorrected distance visual acuity, *CDVA* corrected distance visual acuity, *D* diopter.

### Refraction, predictability, and keratometry

The mean sphere, cylinder, and SEQ values at 6 months postoperatively did not significantly differ between the two groups. The ratio of the mean postoperative SEQ within ± 0.50 D of the intended target was 85% and 70% in the Suction loss and Uneventful groups, respectively. The slope and correlation coefficient for the attempted versus achieved SEQ were 0.9367 and 0.9338 for the Suction loss group and 0.9426 and 0.9181 for the Uneventful group, respectively (Fig. 1). The average postoperative keratometry and mean corneal toricity did not significantly differ between the two groups (Table 2).

### Corneal higher-order aberrations

**Figure 2:**
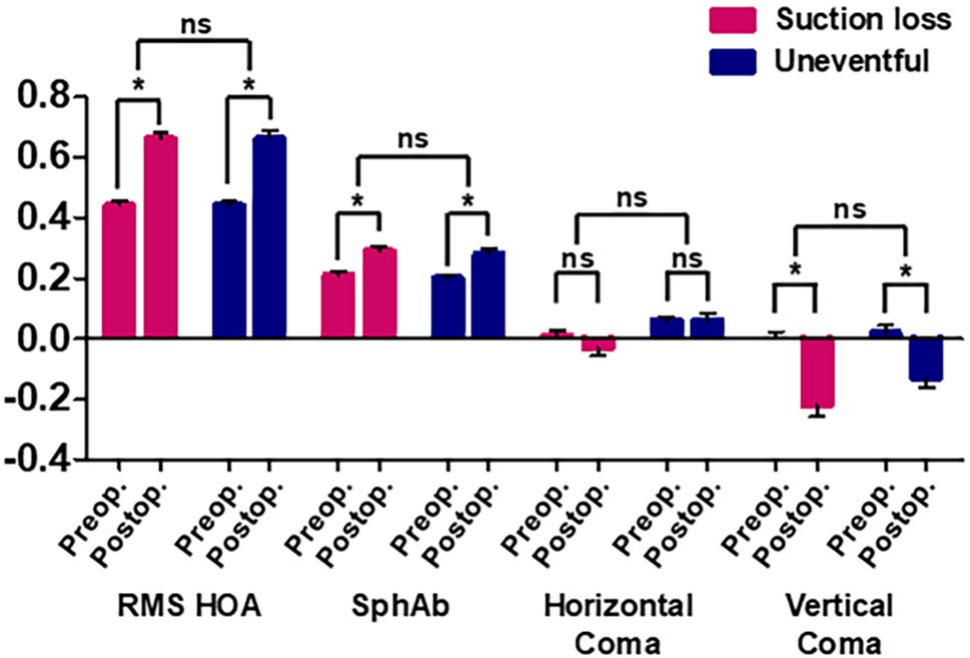
Changes in corneal higher-order aberrations (HOAs) at 6 months after uneventful small-incision lenticule extraction (SMILE) (Uneventful group) and immediate reapplication of the SMILE procedure after suction loss (Suction loss group). Data are presented as the mean ± standard error of the mean (SEM). *RMS* root mean square, *SphAb* spherical aberration, *ns* not significant. *Significant difference.

Table 3 and Fig. 2 present the changes in the corneal wavefront aberrations after surgery. Root mean square values for total higher-order aberrations (HOAs) and spherical aberrations exhibited significant increases at 6 months postoperatively, while the vertical coma values exhibited a significant decrease in both groups. The horizontal coma values did not change significantly after surgery in either group. However, the Suction loss group exhibited significantly lower preoperative and postoperative 6-month horizontal coma values than those of the Uneventful group. Changes in corneal HOAs, including total HOAs, spherical aberration, and horizontal and vertical coma, did not significantly differ between the two groups.

**Table 3:** Comparison of corneal aberrations (μm) of eyes that underwent immediate SMILE reapplication after suction loss and contralateral uneventful SMILE groups.

Corneal aberrations (μm)	Suction loss	Uneventful	*P*
**Root mean square of total higher-order aberrations**			
Preoperative	0.44 ± 0.11 (0.27–0.81)	0.44 ± 0.11 (0.22–0.85)	0.936
6-month postoperative	0.66 ± 0.18 (0.34–1.10)	0.66 ± 0.22 (0.32–1.44)	0.948
*P* (preoperative vs 6-month postoperative)	< 0.001*	< 0.001*	
Δ (preoperative vs 6-month postoperative)	0.23 ± 0.19 (− 0.07 to 0.67)	0.22 ± 0.27 (− 0.08 to 0.89)	0.795
**Spherical aberration**			
Preoperative	0.21 ± 0.09 (0.04 to 0.43)	0.20 ± 0.09 (0.10 to 0.35)	0.500
6-month postoperative	0.29 ± 0.14 (0.02 to 0.56)	0.28 ± 0.15 (− 0.01 to 0.61)	0.676
*P* (preoperative vs 6-month postoperative)	0.002*	0.009*	
Δ (preoperative vs 6-month postoperative)	0.08 ± 0.13 (− 0.19 to 0.39)	0.08 ± 0.16 (− 0.18 to 0.42)	0.973
**Horizontal coma**			
Preoperative	0.01 ± 0.13 (− 0.26 to 0.22)	0.06 ± 0.11 (− 0.14 to 0.26)	0.013*
6-month postoperative	− 0.03 ± 0.22 (− 0.62 to 0.37)	0.06 ± 0.21 (− 0.65 to 0.43)	0.012*
*P* (preoperative vs 6-month postoperative)	0.054	0.809	
Δ (preoperative vs 6-month postoperative)	− 0.05 ± 0.18 (− 0.40 to 0.35)	− 0.01 ± 0.17 (− 0.65 to 0.27)	0.167
**Vertical coma**			
Preoperative	0.00 ± 0.20 (− 0.43 to 0.61)	0.02 ± 0.20 (− 0.39 to 0.67)	0.544
6-month postoperative	− 0.22 ± 0.33 (− 1.00 to 0.74)	− 0.13 ± 0.26 (− 0.64 to 0.38)	0.067
*P* (preoperative vs 6-month postoperative)	< 0.001*	0.001*	
Δ (preoperative vs 6-month postoperative)	− 0.20 ± 0.27 (− 0.61 to 0.54)	− 0.14 ± 0.23 (− 0.67 to 0.39)	0.148

Results are expressed as mean ± standard deviation (range).

Δ = change.

*Significant statistical difference between preoperative and postoperative values.

## Discussion

The present paired-eye study aimed to investigate the surgical outcomes of patients who underwent immediate reapplication of the SMILE procedure without adjustment of the surgical parameters following suction loss and to compare these outcomes with those of contralateral eyes that underwent uneventful SMILE. Our findings revealed that the visual and refractive outcomes were similar between cases of immediate reapplication of SMILE without adjustment of the surgical parameters after suction loss and uneventful cases, regardless of the surgical stage at the time of suction loss. In this study, suction loss occurred during the posterior lenticule surface cut in 53% of eyes and during the cap cut in 47% of eyes. The planned cap thickness ranged from 90 to 135 μm. According to a study by Reinstein et al., treatment options for suction loss during the posterior lenticule surface cut include thinner cap SMILE, thin-flap LASIK, and Re-SMILE^15^. Treatment options for suction loss during the cap cut include continuation of SMILE, re-initiation of the cap cut, and thin-flap LASIK. The treatment options are recommended according to the type of patient eye movement, sustained centration, tracked interface, and planned thickness of the cap. In addition, Liu et al. considered the progression rate of the posterior lenticule surface cut procedure before suction loss when selecting a treatment option^18^. According to Liu et al., LASIK should be recommended when the posterior lenticule surface cut procedure has progressed by > 10% to avoid an irregular posterior lenticule surface and stromal-free slivers. In Liu et al.’s study, the posterior lenticule surface cut or cap cut was repeated in all cases, regardless of the progression rate. In our study, we did not record the progression rate of a particular step before suction loss; therefore, we could not determine the number of cases of suction loss that occurred after > 10% of the posterior lenticular surface was created. However, all suction loss cases treated with immediate repetition of the SMILE technique achieved visual and refractive outcomes comparable to those of the contralateral eyes that underwent uneventful SMILE. Our results suggest that it is possible to achieve clinically safe and effective outcomes by immediate reapplication of the SMILE procedure after suction loss, even if the posterior lenticule surface cut has progressed by > 10%. This may be attributed to the high level of accuracy and reproducibility of the femtosecond laser platform currently used^19^. In addition, treatment centration during the immediate repetition of the SMILE procedure was based on the triple centration markings by which the original treatment center was confirmed. Therefore, treatment centration could be accurate even in the Suction loss group. Other factors such as surgeon experience could also affect clinically comparable outcomes between the 2 groups.

For corneal HOAs, only the horizontal coma values significantly differed between the Suction loss and Uneventful groups at both the preoperative and 6-month postoperative measurements. However, the changes in the horizontal coma values were similar between the two groups. Other HOAs, including the vertical coma, spherical aberration, and root mean square of total HOAs, did not significantly differ between the two groups. All SMILE procedures were performed as originally planned in the Suction loss group. Accordingly, visual and refractive outcomes, including corneal HOAs, did not differ between the two groups.

Surgical complications, such as a double interface layer or torn lenticule, were not observed in either group. No eyes developed intraoperative complications such as lenticule dissection and extraction issues in the Suction loss group. It seems that the femtosecond laser platform can reproduce surgical procedures without causing lenticule-related complications, even in cases of suction loss.

Limitations of our study include the lack of the progression rate records before the suction loss. Especially, suction loss cases during the posterior lenticule surface cut could be biased to early stage. Therefore, it is hard to generalize our results to all suction loss cases at any step. A further study including data of the degree of the ongoing step progression in suction loss cases is needed to generalize our results. In addition, we only investigated results 6 months after the surgery. There could be differences between early postoperative outcomes. However, on the slit lamp examination conducted 1 or 2 days after the surgery, there was no sign of corneal edema, striae or opacity.

In conclusion, immediate reapplication of the SMILE procedure without adjustment of the surgical parameters after suction loss is a clinically safe and an effective option regardless of the surgical stage at the time of suction loss.

## Data Availability

The datasets generated during and/or analysed during the current study are available from the corresponding author on reasonable request.
